# Miniproteins as Phage Display-Scaffolds for Clinical Applications

**DOI:** 10.3390/molecules16032467

**Published:** 2011-03-14

**Authors:** Frederic Zoller, Uwe Haberkorn, Walter Mier

**Affiliations:** 1 Clinical Cooperation Unit Nuclear Medicine, German Cancer Research Center, INF 280, 69120 Heidelberg, Germany; E-Mails: frederic.zoller@med.uni-heidelberg.de (F.Z.); uwe.haberkorn@med.uni-heidelberg.de (U.H.); 2 Department of Nuclear Medicine, University Hospital Heidelberg, INF 400, 69120 Heidelberg, Germany

**Keywords:** phage display, miniprotein, scaffold, *in vivo* diagnostics, protein engineering

## Abstract

Miniproteins are currently developed as alternative, non-immunoglobin proteins for the generation of novel binding motifs. Miniproteins are rigid scaffolds that are stabilised by alpha-helices, beta-sheets and disulfide-constrained secondary structural elements. They are tolerant to multiple amino acid substitutions, which allow for the integration of a randomised affinity function into the stably folded framework. These properties classify miniprotein scaffolds as promising tools for lead structure generation using phage display technologies. Owing to their high enzymatic resistance and structural stability, miniproteins are ideal templates to display binding epitopes for medical applications *in vivo*. This review summarises the characteristics and the engineering of miniproteins as a novel class of scaffolds to generate of alternative binding agents using phage display screening. Moreover, recent developments for therapeutic and especially diagnostic applications of miniproteins are reviewed.

## 1. Introduction

Over the last two decades, monoclonal and polyclonal antibodies have become an important class of binding molecules for biomedical applications. Their *in vivo* generation by the immune system (first generation antibodies) or *in vitro* by synthetic immunoglobulin repertoires (second generation antibodies) facilitates the selection of highly specific and tight-binding affinity reagents with a widespread molecular diversity [1]. Immunoglobulin (Ig) molecules offer the entire spectrum of defined molecular recognition and interaction with proteins, peptides, sugars and diverse small molecules. 

However, the Ig format has a number of limitations which restricts its use in therapeutic applications [2]. For example, the molecular architecture of antibodies, with disulfide bridges and complex glycosylation patterns, requires complex cost-intensive biotechnological manufacturing processes, compared to the synthesis of peptides or small molecules. The large size of such bivalent, multidomain proteins can induce immunogenic side effects [3], increase serum half-life and reduce the rate of tissue penetration and cell permeability [4]. The targeting of antibodies is primarily limited to extracellular binding sites such as cell-membrane receptors. Furthermore, the pharmacokinetic properties of antibodies, in particular the slow blood pool clearance, disqualify antibodies from diagnostic use. 

**Figure 1:**
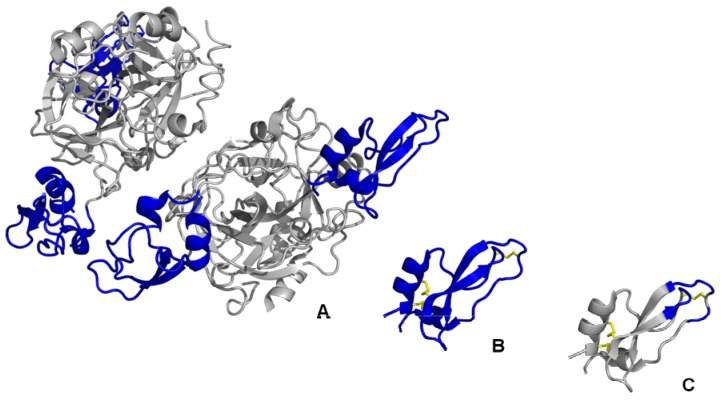
The miniaturisation approach. Downsizing of naturally occurring large protein structures (A), such as the Kunitz-type proteinase inhibitor boophilin (PDB ID: 2ODY), via the isolation of functional protein domains (B) leads to miniature protein scaffolds (C) suitable for engineering for phage display library construction. The functional domains are coloured in blue, disulfide bridges are yellow. Structural representations were created using PyMol software [14].

Current research efforts are focused on the miniaturisation of antibodies into smaller formats, adapting the unique target specificity and exceeding their limited application at the same time. In the context of conventional Ig, only a minor part of the molecule is involved in the binding interaction. Therefore, the continuous downsizing of antibodies, enzymes or other large proteins in order to accentuate the binding domain, give rise to a future-perspective approach for the design of alternative binding specificities (Figure 1). Historically, this step helped to understand the relationship between protein size and pharmacological properties. Several antibody fragments, such as the antigen-binding fragment (F_ab_) or the single-chain variable fragment (scFv), were engineered to act as non-immunogenic targeting proteins with improved biodistribution and blood clearance properties resulting from minimizing their size [5]. These so-called domain antibodies (dAbs) or nanobodies have already been designed to facilitate therapeutic as well as diagnostic applications [6,7]. 

To overcome these limitations, an alternative strategy is to engineer non-immunoglobulin proteins which incorporate a customised affinity domain. Consequently, protein scaffolds are of growing interest as an alternative to antibodies for molecular recognition [8]. Nevertheless, the combinatorial diversity, which is the benchmark of the antibodies, has to be ensured. This basic requirement can be verified by using a protein library construct that consists of a protein framework, displaying variable regions with diversified patterns assuming the role of the complementarity determining regions (CDRs) in the antibody [1]. 

The use of functional miniature proteins based on naturally occurring templates or on rational constructs represents a promising alternative approach to antibody miniaturisation. These so-called miniproteins are defined as stably folded polypeptides which form a rigid framework, featuring protein-like properties and are accessible via chemical synthesis. The general idea of the miniprotein scaffold concept is the integration of an affinity function into a well-defined, stably folded structural framework by locally reshaping the molecular surface through primary structure modifications [9]. The ultimate goal of this approach is the design of small molecule mimetics. 

Within this context, molecular selection techniques, such as phage display [10,11] allow for the combination of the miniprotein scaffold concept with the design of random protein libraries, where the affinity function is displayed on an accessible surface region of the scaffold. Stringent selection protocols enable the generation of binding specificity against virtually any given target. This approach is also applicable to related high throughput screening (HTS) technologies such as yeast two-hybrid systems [12] or ribosome display [13]. To date, commercially available phage display libraries are restricted to small peptides of 8–12 amino acids, which show a relatively low stability *in vivo*. To overcome this problem, phage display-derived miniprotein engineering facilitates the generation of novel binding entities for applications *in vivo*. 

This review highlights the properties and engineering efforts of miniprotein scaffolds as a novel class of alternative binding motifs and their application in phage display screening for the development of novel non-immunoglobulin binding reagents. In addition, recent progress in the development of miniproteins for therapeutic and diagnostic applications will also be reviewed.

## 2. Properties of Miniprotein Scaffolds

The design and selection of suitable miniproteins for phage display screening have to meet several requirements. In general, the design of miniprotein constructs can be based either on previously characterised three-dimensional topologies or on *de novo* engineered scaffolds. The properties that are crucial for scaffolds and their application for phage display are:

(i) The monomeric, small polypeptide chain has to be highly stable against enzymatic degradation, easy to engineer, and efficiently produced by recombinant expression or solid-phase peptide synthesis;
(ii) The formation of a defined, rigid three-dimensional topology by secondary structural elements is mandatory;
(iii) The tolerance to sequence variations or insertions within the recognition site has to conserve the protein folding or stability;
(iv) The accessibility to a surface domain or binding pocket as a recognition site has to be ensured;
(iv) A well-defined hydrophobic core that contributes to the high free energy of folding is advantageous.

An optimal scaffold should include all these characteristics to ensure that the variable binding epitope is exposed upon the surface structures, formed from α-helices, β-sheet or β-turn motifs. These essential secondary structural elements lead to a rigid protein-like architecture, even within small polypeptides with less then 30 amino acids, and constitute a high proteolytic stability, which can be further increased by disulfide linkages. Nevertheless, the accommodation of the randomised binding epitope within the protein template has to be achieved without changing the overall topology and folding capability of the scaffold. 

### 2.1. Advantages of miniprotein scaffolds

The key benefits of miniprotein binders are the pharmacokinetic properties that result from their small size. The remarkable *in vivo* stability in combination with a high binding affinity and selectivity make these scaffolds excellent candidates for diagnostic applications [15]. The superb pharmacokinetic properties allow a rapid accumulation in the region of interest, provide a fast clearance from non-specific compartments and therefore enable precise targeting *in vivo* [16]. These aspects have already been addressed in a number of studies on the downsizing of antibodies and their engineered fragments to improve their therapeutic and diagnostic potential [17,18]. Recently, molecular imaging tools, such as single photon emission tomography (SPECT) or positron emission tomography (PET) have gained importance in the preclinical evaluation of pharmacokinetic properties of novel drugs. These modalities enable a non-invasive visualisation of biological processes at the molecular level. In clinical oncology, the combination of molecular imaging modalities with anatomic imaging techniques, such as computed tomography (CT) or magnetic resonance imaging (MRI), is of upmost significance. These techniques facilitate the detection of pathophysiological processes at a curable stage and allow an optimised therapy plan as well as the detailed monitoring of its response [19]. In this regard, phage display-evolved miniature protein binders, which specifically target tumour-associated antigens, have substantial potential for radionuclide imaging [15,20] and peptide receptor radiation therapy [21,22]. 

In addition to these advantages described, the structural rigidity of miniprotein scaffolds facilitate an optimised shape complementarity to the recognition binding site(s) and to a minimal loss of conformational entropy upon formation of the ligand-target-complex. The interaction of the hydrophobic exterior surface of the scaffold with the targeted receptor causes the release of water molecules from the binding interface. These thermodynamic aspects result in a higher overall Gibbs free enthalpy for the recognition process and results in a higher binding affinity of the scaffold [23]. In the case of linear peptides, where the underlying mechanism is an induced fit, the entropic cost of taking a specific geometry has to be considered. 

Miniproteins mimic the features of large proteins, show antibody-like properties, but cause less toxic or immunogenic side effects and can be produced by less-complex and inexpensive means. For industrial-scale production, solid-phase peptide synthesis techniques enable a very reliable, inexpensive alternative to biotechnological manufacturing routes. These fundamental aspects highlight the potential of phage display-derived alternative scaffolds for the generation of new molecular entities and raise great expectations for pharmaceuticals companies.

### 2.2. Classification of miniprotein scaffolds

**Figure 2:**
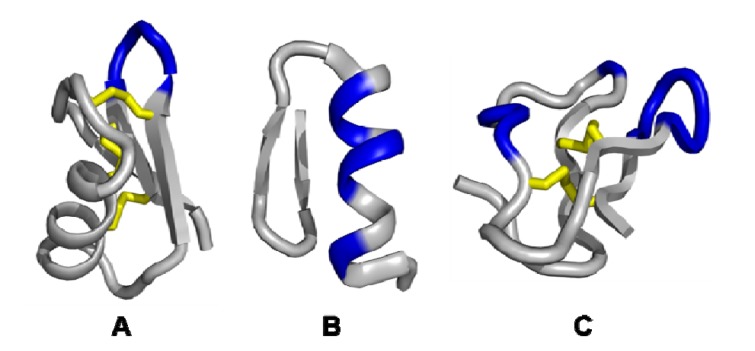
Schematic representations of different scaffold categories. The first type of scaffold displays a single exposed loop embedded in a ridge template (A). The second scaffold class contains a non-contiguous surface region formed by secondary structural elements (B). The third category shows a complex backbone architecture which encloses multiple discontinuous variable loop segments forming a coherent interface (C). Variable sequence regions are coloured in blue, disulfide bridges are coloured in yellow. The schematic representations were generated from the RCSB Protein Data Base: (A): Scorpion charybdotoxin (PDB ID: 2CRD); (B): Single zinc finger DNA-binding domain (PDB ID: 1ZNF); (C): Cellulose binding domain from cellobiohydrolase CeI7A (PDB ID: 1CBH). Structural representations were illustrated using PyMol software [14].

The structural requirements discussed restrain the pool of suitable scaffold templates. Based on the peptide backbone, miniprotein scaffolds can be classified into three groups (Figure 2). 

The first type of miniprotein scaffolds displays a single loop on the surface of the rigide template, primarily formed by hydrophobic patches or β-turn elements. The second class contains a non-contiguous, but composed surface region that is protruded by secondary structural elements. These elementary motifs are rigid, densely packed and reveal a large recognition interface shaped by mostly non-polar, steric complementary dispositions. The third category exhibits multiple non-continuous variable loop segments which form a coherent interface enclosed by a constrained backbone architecture. This structure facilitates a multi-point attachment to the target with high affinity and specificity. A well-positioned geometry is provided by the Ig class [24].

The selection criterion for a miniprotein scaffold is dependent on the number of variable amino acids within the random sequence and the corresponding variability of the protein library. However, the overall topology of the template and spatial alignment of the displayed random segment has to match to the binding site of the selected target. This aspect could require information about the target structure, which is often unknown in the early stages of development. However, the ultimate criterion for identifying of a novel protein-based binding agent is the structure-affinity relationship between the engineered scaffold and the molecular shape of the intended target.

### 2.3. Scaffold library design for phage display screening

The selection and design of miniprotein templates for phage display library construction and their subsequent screening relies on several parameters. A general requirement is the translation of the genotype of the bacteriophage into the corresponding phenotype, which represents the resultant functional peptide. The first step is the generation of a random peptide sequence at the genetic level. The diversification is achieved by using chemically synthesised oligonucleotides encoding for the individual amino acids to generate the combinatorial repertoire [25]. In this context, the use of codon-corrected trinucleotide building blocks was validated as the most efficient method providing maximal sequence diversity compared to NNK or NNS nucleotide mixtures [26,27]. 

In scaffold library design, the positions of variable amino acids are strictly allocated to the recognition site within the peptide template. This is verified by random insertion of trinucleotide codons into the oligonucleotide sequence. The introduction of the scaffold library into the phage genome is accomplished by fusing a randomised oligonucleotide sequence to a phage coat protein. In the biopanning cycles, the pool of phages representing the library is screened against immobilised targets. Specific binders are isolated, amplified in bacteria and identified by DNA-sequencing.

In order to ensure that a properly folded and functional miniature protein is displayed, post translational modifications by the expression host should be kept to a minimum [28]. The random peptides displayed may not represent the optimal sequence for accurate folding and stability. The installation of a spacer between the scaffold and the coat protein can assist the folding procedure. In the case of cystine-constrained miniproteins, the expression host and the selection environment have to be properly designed to permit correct disulfide formation [29]. The filamentous M13-fusion phage has become the most reliable host system for displaying functional peptides and proteins on its surface. This system was proven in practice to be useful for disulfide-constrained miniproteins [30] and engineered scaffolds [31] with complex secondary structures [32].

### 2.4. Miniprotein engineering

The synthetic access to the screening hit can be achieved by either a biotechnological or chemical approach. The evaluation of the pharmacological properties of the identified binding domain usually requires the production of milligram amounts of the properly folded peptide. In contrast to the biotechnological production routes that are used for antibodies, combinatorial protein chemistry, such as solid-phase peptide synthesis (SPPS) represents a reliable, inexpensive, and fully automated technique for generating the primary structure of peptides [33]. The chemical synthesis guarantees the most direct route to the individual peptide, which is the best control over a subsequent tertiary structure formation. Moreover, SPPS allows for the integration of both proteinogenic and non-proteinogenic amino acid building blocks for the defined lead structure optimisation. The total chemical synthesis of larger proteins from SPPS-derived peptides is feasible using chemoselective ligation strategies, where native chemical ligation (NCL) has become the method of choice [34]. Importantly, a complete chemical synthesis highly simplifies the development of a GMP-compliant production, which is crucial for introduction of new molecular entities into clinical practice.

Chemical peptide synthesis also enables the specific modification of almost any amino acid within the protein [35]. Owing to their remarkable structural, chemical and thermal stability, miniproteins can tolerate a high degree of chemical modifications. For the pharmacological evaluation of novel binding entities, the labelling with fluorescent dyes or biotinylation are the established derivatisation for *in vitro* experiments. The conjugation with bifunctional metal-chelating agents, such as polyaza-cycloalkanes [36] enables both *in vitro* and *in vivo* studies with radionuclides. In particular, the ε-amino function of lysine or the thiol function of cysteine is a key position for the connection of appropriate labelling units with the peptide backbone. The tyrosine moiety facilitates a simple and selective radiolabelling with radioactive iodine isotopes [37].

The chain assembly of the linear peptide precursor via SPPS is, however, just the initial step of the entire engineering process. Protein folding is a cooperative event caused by non-covalent interactions such as hydrogen bonds, charge-charge and especially hydrophobic interactions. Thus, the translation of the amino acid sequence into the correct structure and function has to be ensured. The controlled formation of protein-like properties via secondary or tertiary structures is a challenging process in the transfer from the phage to the isolated peptide. The understanding of the relationship between sequence and structure is obtained from the analysis of well-characterised proteins [38]. Several bioanalytical and biophysical methods, such as mass spectrometry, X-ray crystallography, NMR and CD spectroscopy as well as computational molecular modelling are essential in protein and peptide design.

Basically, the adaption of natural protein folds and their imitation is the route to success for downsizing antibodies, enzymes or other large proteins in order to engineer functional miniature proteins. In general, there are two strategies to approach this task; first, the construction of novel macromolecules by exploiting the interacting functions of existing natural bioactive protein structure; and second, the rational design via reassembling exposed secondary structural elements into a constrained template with well-defined folds [39]. An alternative rational approach is the molecular grafting of a pre-organised functional unit onto a natural, rigid framework to combine the high stability of the protein scaffold and the bioactivity of the grafted template. This strategy might also be feasible for the stabilisation of short linear, phage display-derived peptides. 

## 3. Examples of Miniprotein Display Scaffolds

During the last two decades, versatile protein scaffolds were investigated for the construction of phage display libraries to identify alternative binding agents. They were designed for screening protein domains, ranging from only 23 residues [40] up to several hundred amino acids [41,42]. Both artificial constructs and native protein structures, incorporating alpha-helical, beta-barrel or cystine-constrained structural elements, were exploited as lead compounds for appropriate miniproteins. The main source for miniprotein scaffolds are naturally occurring structures from plants, reptiles and microorganisms (Table 1). The most promising candidates are endogenous protease inhibitor proteins. This class of proteins provides exposed loop structures as active site, which are highly stable and appropriate for mutagenesis [43]. Protease inhibitors, such as cyclotides and other knottins, exhibit an advantageous structural topology, are remarkable stable and tolerant to sequence variations, which classify these protein-like peptides as ideal display scaffolds. 

Recent proceedings demonstrate the ability of engineered miniproteins as alternative scaffolds for medical applications. Knottins and Affibodies are of outmost interest in this context. As already mentioned, the size of the scaffold limits not only their application for diagnostic purposes, but also their synthesis by peptide chemistry. In this section, miniprotein display scaffolds, which are suitable for phage display library construction and applicable as alternative binding reagents are discussed. Moreover, their use as *in vivo* diagnostic imaging agent is reviewed in particular.

**Table 1:** Examples of suitable miniprotein scaffolds for phage display library construction. Scaffold categories: Scaffolds displaying a single exposed loop (A); scaffolds displaying a non-contiguous surface domain (B); scaffold displaying multiple discontinuous variable regions (C). Abbreviations: *Ecballium elaterium* trypsin inhibitor II (EETI-II); sunflower trypsin inhibitor (SFTI-I); cellulose binding domain (CDB); ecallantide plasma kallikrein inhibitor (DX-88); cystine-stabilised β-sheet (CSB); disulfide bridge (SS); amino acids (aa); monoclonal antibodies (MAbs).

Scaffold name	Acronym	Scaffold Category	Secondary structure motifs	Size (aa)	Random positions	Origin	Binding specificity (target)	References
EETI-II	Cyclotide	A	CSB / 3 SS	28	6 aa / loop	Plant (*Ecaballium elaterium*)	Chymotrypsin, trypsin, integrins	[44,45]
Min-23	Knottin	A	CSB / 2 SS	23	8 -10 aa / β-turn	Rational design	MAbs, HIV-1 Nef, Tom70, AMA-1	[40]
Scorpion toxin	Knottin	A	CSB / 3 SS	37	4 aa / loop	Scorpion (*Leiurus quinquestriatus hebraeus*)	Acetylcholin receptor, MAbs	[46,47]
SFTI-I	Knottin	A	circular, 1 SS, 2 β-sheets	14	6 -8 aa / loop	Plant (*Helianthus annuus*)	Trysin, chymotrypsin	[48,49]
Z domain	Affibody	B	3 α-helical bundle	58	13 aa / 2 α-helices	Bacteria (*Staphylococcus aureus*)	Taq polymerase, Her-2/neu, CD28	[20,50,51]
Zinc finger	-	B	α-helix / β-sheet / Zn^2+^	26	5 aa / α-helix	Frog (X*enopus laevis)*	MAbs	[52]
CBD	Knottin	C	CSB / 3 SS	36	11 + 7 aa / 3 loops	Fungus (*Trichoderma reesei*)	Alkaline phospathase, α-amylase	[53,54]
DX-88	Kunitz domain	C	α-helix / 2 β-sheets / 3 SS	58	5 + 4 aa / 2 loops	Human *(type VI collagen*)	Plasma kalikrein	[32,55,56,57]
Tendamistat	Knottin	C	β-sandwich / 2 SS	74	3 + 3 + 6 aa / 3loops	Bacteria (*Streptomyces tendae*)	α-Amylase, MAbs, integrins	[58,59]

### 3.1. Single exposed loop scaffolds

The most representative scaffolds displaying a single, variable loop structures belong to the knottin family. These small protein-like peptides, some of which act as protease inhibitors are comprised of 23–37 amino acids and have a well-arranged knotted topology that is formed by disulfide bonds. The disulfide-constrained core confers an outstanding thermal, chemical and proteolytic stability and was proven to be non-immunogenic [60]. Owing to their natural function as host defence agents, knottins feature high intrinsic and antagonistic activities. This makes them an ideal tool for drug design [61], molecular diagnostics [15] and endoradiotherapy [21,22]. For several *in vitro* screening technologies, including phage display, they were employed as a molecular template for the construction of constrained repertoires [53,54]. The expansion of the protease-binding site by substitution with several random amino acids and subsequent selection against different molecular targets resulted in several novel binding reagents [44,62]. 

**Figure 3:**
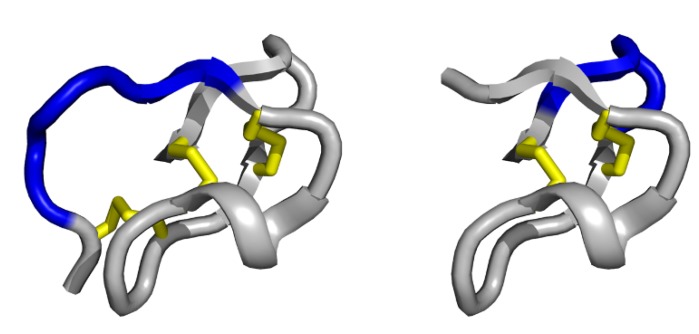
Schematic representation of *Ecballium elaterium trypsin inhibitor II* (left) and its functional miniature scaffold Min-23 (right), showing the cystine-stabilised β-sheet (CSB) motif of the knottin family (disulfide bridges are coloured in yellow). The single exposed variable loop (coloured in blue) is permissive to the variation of six amino acids in EETI-II and 8 to 10 in Min-23, respectively. Structural representations (PDB ID: 2IT7) were illustrated using PyMol software [14].

The trypsin inhibitor *Ecballium elaterium* (EETI-II), a squash-type inhibitor isolated from squirting cucumber seeds, is the prototype of this miniprotein family [63]. EETI-II consists of 28 amino acids with six conserved cysteine residues, shaping the cystine-stabilised β-sheet (CSB) motif (Figure 3) [64,65]. The CSB is composed of a triple-stranded β-sheet stabilised by two disulfide bridges and is found as the elementary structural pattern in nearly 50% of all known small disulfide-rich protein families [66]. Remarkably, EETI-II has the ability to configure all of its disulfide bonds correctly and to fold efficiently *in vitro*, which facilitates its chemical synthesis [67]*.* Recent molecular grafting studies have demonstrated the tolerance of the exposed loop for the integration of defined binding domains [68]. Knottin scaffold binders with nanomolar affinity against the α_v_β_3_ integrin, a receptor overexpressed in angiogenetic tumours, were identified by screening yeast-displayed libraries containing the binding motif Arg-Gly-Asp (RGD) with random flanking residues [69]. Moreover, PET imaging studies of tumour neovascularisation using these radiolabelled knottin peptides exhibit a more favourable tissue distribution in terms of an increased tumour uptake and a decreased nonspecific liver uptake compared to the benchmark, cyclic RGD-pentapeptide c(RGDyK) [70]. An improved clearance of the knottin tracer results in a higher tumour to background ratio compared to the gold standard ^18^F-fluorodesoxyglucose (FGD). This was demonstrated in lung tumour-bearing mouse model [71].

A rational design approach focussed on downsizing EETI-II by *N*-terminal truncation, resulted in the development of a stably folded 23-residue peptide. This scaffold named Min-23 comprises only two disulfide bridges instead of three found in the parent protein. Min-23 was shown to fold in a native-like manner caused by the cystine-stabilised β-sheets (CSB) motif, which encloses a stable autonomous folding unit (Figure 3). While this motif is devoid of any naturally occurring active site, it was shown to be highly permissive to substitutions of 8 to 10 amino acids within a single exposed β-turn on its surface. These characteristics classify Min-23 as a suitable peptide scaffold for phage display library construction or molecular grafting of recognition sites to engineer novel biological specificities. Peptide epitopes from hemagglutinin or the Gla-protein were integrated on the exposed loop. Also, randomised sequences of Min-23 were constructed using a phage library and were screened against different targets, resulting in the isolation of novel specificities [40].

Among the knottin family, the sunflower trypsin inhibitor I (SFTI-I) is the smallest reported ribosomally synthesised cyclic peptide. SFTI-I is a head-to-tail cyclic, disulfide-constrained 14-mer peptide extracted from sunflower seeds and possesses an exceptionally high trypsin-inhibitory activity [72]. Owing to its convenient solid-phase peptide synthesis and proteolytic stability, this knottin is a promising candidate for peptide-based drug design. Recently, *in vivo* studies using radiolabelled SFTI derivatives demonstrate beneficial pharmacokinetic properties for molecular diagnostic applications [48].

Despite these developments, knottin peptide chemistry is a challenging discipline caused by the equilibration between the formation of intra- and inter-molecular disulfide bridges. The intramolecular oxidation often results in the formation of dimers or oligomers and result in low overall chemical yields. In the case of multiple disulfide formations, complex structure determination experiments such as NMR spectroscopy are required as to prove the native disulfide connectivity. In terms of high throughput screening, these drawbacks can affect the progression in the development of novel molecular entities.

### 3.2. Non-contiguous surface domain scaffolds

**Figure 4:**
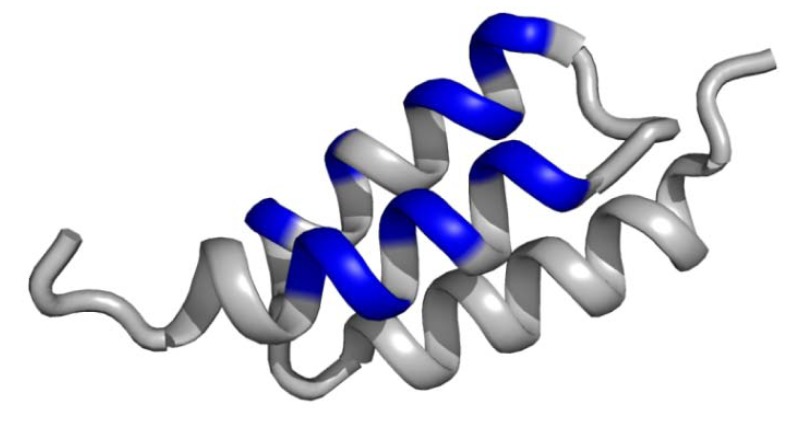
Schematic representation of the Z domain scaffold, termed Affibody, derived from the B-domain of the *Staphylococcal aureus* immunoglobulin-binding protein A. The composed surface formed by the three-α-helical structure (coloured in blue) presents 13 variable amino acid positions in two α-helices for combinatorial randomisation via phage display. The structural data (PDB ID: 2B89) were illustrated using PyMol software [14].

Composed surfaces, formed by helical coiled coils, helix bundles or β-sheets belong are the most abundant structural domains in proteins. The most important display scaffold in this category is the so-called Affibody, which was originally derived from the B-domain of *Staphylococcal aureus* immunoglobulin-binding protein A. Selected mutations at key positions of the B-domain revealed an optimised version of the properly folded scaffold, termed the Z-domain [73]. This 58 amino acid peptide consists of a cysteine-free, three-α-helical bundle with high thermal and proteolytic stability as well as high suitably for sequence mutagenesis. The Z-domain was one of the first non-β-sheet peptide backbones used for combinatorial library construction for phage display [74]. The combinatorial library construction with 13 random amino acid positions within two helices (Figure 4) has generated specific binders against a variety of different targets, such as tumour necrosis factor-α, IL-8, gp120, CD28, HER2 and EGFR, with affinities in micromolar to the picomolar range [75]. Access of such binders is feasible both by recombinant production [76] and solid-phase peptide chemistry [77,78]. 

Affibody molecules gained importance rapidly for protein engineering for applications in life science [50]. Among alternative scaffold proteins, this class of miniproteins was studied intensely as a candidate for cancer imaging [20,79]. The first Affibody ligands for medical imaging were selected by phage display against the HER2, a transmembrane tyrosine kinase receptor belonging to the human epidermal growth factor receptor family [80]. The over-expression of HER2/neu receptor was detected in a number of malignant tumours, such as breast, ovary and bladder carcinomas. Versatile radiolabelling approaches were conducted to demonstrate the tumour targeting properties of the picomolar affine Affibody Z_Her2:342 _in xenograft models [81,82,83,84,85]. Recent pilot studies in humans have also exhibited its applicability for breast cancer PET/CT imaging [86]. This tracer was also shown to be relevant for monitoring of trastuzumab therapy, a monoclonal antibody blocking HER2 [87,88]. 

Another miniprotein in this scaffold category is the zinc-finger motif. This 26-residue peptide was used to generate phage display libraries by mutagenesis of five amino acids in the α-helical portion. This zinc finger scaffold was used as a conformational template for the selection-driven design of peptidomimetics [52]. Larger protein families, such as repeat-motif peptides, were built from repetitive structural units of elongated domains with variable target-binding surfaces. Combinatorial libraries of designed ankyrin repeat proteins (DARPins) [89] and leucine-rich repeat (LRR) [90] were generated to evolve novel binding specificities. However, the large size of repeat polypeptides probably excludes the application for diagnostic purposes and limits access via chemical peptide synthesis.

### 3.3. Multiple discontinuous domain scaffolds

**Figure 5:**
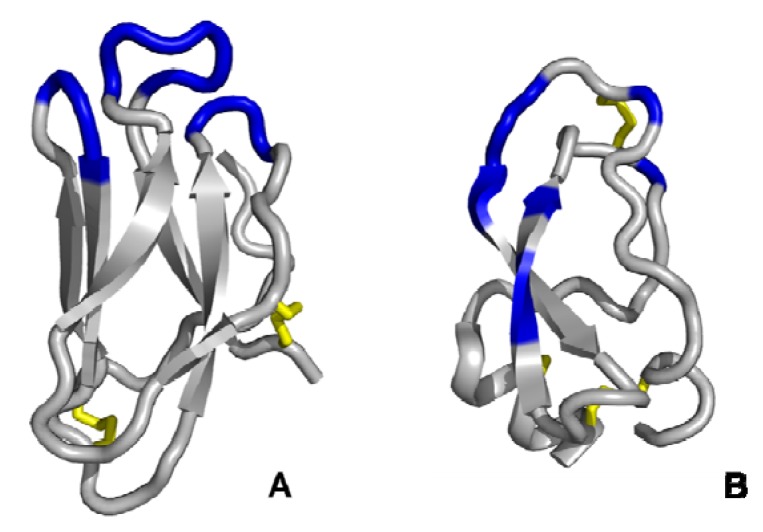
Schematic representations of tendamistat (A) and the Kunitz-domain scaffold (B). The discontinuous surface domains form a coherent interface for the target interaction (coloured in blue, disulfide bridges are coloured in yellow). The structural data (PDB ID: (A): 1OK0; (B): 1AAP) were illustrated using PyMol software [14].

Scaffolds displaying multiple non-continuous variable domains facilitate an increased affinity compared to a single domain scaffolds. The complementary geometry results in multi-point attachment, in which only one loop can interact with the active site, while the others ensure a close contact with regions distal to it. The structurally conserved framework is dominated by β-sandwiches formed by two antiparallel β-sheets exposing the variable sequence pattern in the β-turn shaped. This geometry is exemplary shown for the tendamistat scaffold (Figure 5).

Tendamistat is a 74-residue inhibitor derived from α-amylase from *Streptomyces tendae*. Its conserved structure accommodates two disulfide bonds and five β-sheet motifs enclosing three variable loop segments. The tolerance to sequence randomisation in loop II and III was demonstrated by phage display library construction [59]. Random-flanking mutagenesis, using libraries containing the RDG motif in loop I, was performed to select binders against integrins [58].

An outstanding example, highlighting the potential of the phage display technology in the development of viable small protein drugs, is the Kunitz domain inhibitor family. This scaffold comprises irregular secondary structures constrained by three disulfide bonds and, exposes multiple variable domains (Figure 5). Its first application was the modelling of novel specificities against protease targets such as aprotinin (bovine pancreatic trypsin inhibitor, BPTI), Alzheimer's amyloid β-protein precursor (APPI), and the tissue factor pathway inhibitor (TFPI) [32,91].

In 2009, the drug ecallantide, a plasma kallikrein inhibitor (KALBITOR^®^, investigation code DX-88, Dyax Corp/Genzyme Corp) was approved by the U.S. Food and Drug Administration as the first-in-class inhibitor for the treatment of acute attacks of hereditary angioedema (HAE), a life-threatening disorder caused by the genetic deficiency of C1 esterase inhibitor [92]. Moreover, it was shown to prevent blood loss in cardiothoracic surgery [93]. DX-88 is a 60-residue miniprotein and was isolated from a Kunitz domain phage display library based on the human lipoprotein-associated coagulation inhibitor (LACI), also known as tissue factor pathway inhibitor (TFPI) [94]. The primary sequences of ecallantide and the wild-type LACI are highly homologous and differ in only seven amino acids [56,57]. It was confirmed to be highly potent and to specifically inhibit plasma kallikrein [43]. An efficient recombinant route of production was established in a yeast host system (*Pichia pastoris*). Employing an analogous strategy, the proteolysis-resistant inhibitor of human neutrophil elastase, DX-890 (EPIhNE4) is currently under investigation as an anti-inflammatory drug for treatment of cystic fibrosis [95].

## 4. Conclusions

Phage display techniques were shown to be valuable research tools in both basic and applied science. As a high-throughput screening platform, it is of high significance for pharmaceutical companies in the screening and development of novel, patentable molecular entities. Nevertheless, the combinatorial library construction [96], which has to fulfil the structural requirements of the scaffold, is only the initial step to arrive at novel binding molecular entities. However, following the screening process, the iterative protein engineering of the identified screening hits and the evaluation of their pharmacological properties are individual milestones of the entire development process.

Engineered miniprotein scaffolds as library templates for display screening technologies offer the potential to overcome the limitations of conventional antibodies. They already entered clinical practise and will be of fundamental importance in therapy and molecular diagnostic in the future. The recent improvements highlight the capacity of these next-generation protein formats for the discovery and development of alternative, non-immunoglobin reagents. Thus, it might be just a question of time, until further phage display-evolved peptides or proteins will contribute to clinical treatment.
